# Dependency and frailty in the older haemodialysis patient

**DOI:** 10.1186/s12877-024-04973-8

**Published:** 2024-05-10

**Authors:** M Pereira, M L Sanchez Tocino, Sebastian Mas-Fontao, P Manso, M Burgos, D Carneiro, A Ortiz, M D Arenas, E González-Parra

**Affiliations:** 1 Fundación Renal Íñigo Álvarez de Toledo, Madrid, 28003 Spain; 2 Facultad de Enfermería, Universidad de Salamanca, Salamanca, Spain; 3 Instituto de Investigación Sanitaria Fundación Jimenez Díaz, Madrid, 28040 Spain; 4 Centro de Investigación Biomédica en Red de Diabetes y Enfermedades Metabólicas Asociadas (CIBERDEM), Madrid, Spain; 5 Servicio de Nefrología, Fundación Jiménez Díaz, Madrid, Spain; 6 Facultad de Medicina y Biomedicina, Universidad Alfonso X, Villanueva de la Cañada, Spain

**Keywords:** Hemodialysis, Aging, Sarcopenia, Dependency, Frailty

## Abstract

**Background:**

Frailty among older adults undergoing hemodialysis is increasingly prevalent, significantly impacting cognitive function, mobility, and social engagement. This study focuses on the clinical profiles of very older adults in hemodialysis, particularly examining the interplay of dependency and frailty, and their influence on dialysis regimens.

**Methods:**

In this observational, descriptive study, 107 patients aged over 75 from four outpatient centers and one hospital unit were examined over a year. Patient data encompassed sociodemographic factors, dialysis specifics, analytical outcomes, lifestyle elements, and self-reported post-treatment fatigue. Malnutrition-inflammation scale was used to measure the Nutritional status; MIS scale for malnutrition-inflammation, Barthel index for dependency, Charlson comorbidity index; FRIED scale for frailty and the SF12 quality of life measure.

**Results:**

The study unveiled that a substantial number of older adults on hemodialysis faced malnutrition (55%), dependency (21%), frailty (46%), and diminished quality of life (57%). Patients with dependency were distinctively marked by higher comorbidity, severe malnutrition, enhanced frailty, nursing home residency, dependency on ambulance transportation, and significantly limited mobility, with 77% unable to walk. Notably, 56% of participants experienced considerable post-dialysis fatigue, correlating with higher comorbidity, increased dependency, and poorer quality of life. Despite varying clinical conditions, dialysis patterns were consistent across the patient cohort.

**Conclusions:**

The older adult cohort, averaging over four years on hemodialysis, exhibited high rates of comorbidity, frailty, and dependency, necessitating substantial support in transport and living arrangements. A third of these patients lacked residual urine output, yet their dialysis regimen mirrored those with preserved output. The study underscores the imperative for tailored therapeutic strategies to mitigate dependency, preserve residual renal function, and alleviate post-dialysis fatigue, ultimately enhancing the physical quality of life for these patients.

**Supplementary Information:**

The online version contains supplementary material available at 10.1186/s12877-024-04973-8.

## Background

Frailty is a multidimensional syndrome characterized by the loss of lean body mass (sarcopenia), weakness, reduced exercise endurance, and decreased activity response to stress. This condition leads to a vicious cycle of functional decline and increased mortality risk [1].

Approximately 42% of adult hemodialysis patients experience frailty, with the prevalence notably increasing in elderly patients [2]. Frailty is associated with a significantly increased risk of mortality and hospitalizations, independent of age and comorbidities [3]. The consequences of frailty in older patients’ dialysis patients include falls, hospitalizations, dependence, reduced quality of life, increased healthcare costs, and mortality.

The number of older patients on hemodialysis has been increasing, including frail individuals [4]. The coincidence of advanced age and accelerated aging in chronic kidney disease (CKD) may explain the high prevalence of frailty in this population [5].

A comprehensive approach to older patients’ on dialysis requires a multidisciplinary assessment encompassing medical, psychosocial, functional, environmental, socio-health, and family factors. In this context, the patient’s gender plays a significant role, as frailty, dependency, and quality of life are all influenced by this variable [6]. Evaluating the patient’s functional, cognitive, nutritional, and psychological status is crucial for establishing an appropriate therapeutic plan that may involve dialysis or alternative options [7].

Frailty in old age can be associated with cognitive impairment, immobility, motor dysfunction, incontinence, and reduced family and social engagement [8]. These factors present logistical challenges in achieving adequate dialysis, as the patient’s ability to engage in dialysis care is diminished, particularly with longer and more frequent sessions [9].

Dialysis has shown to prolong longevity in older patients compared to conservative treatment, but its benefits are limited in the most severe cases [10]. The initiation of dialysis in very older patients CKD patients often leads to a loss of independence and increased dependency [11], with a significant proportion requiring caregiver support or nursing home admission. However, rehabilitative care can potentially reverse this loss of independence [8].

Initiating dialysis in frail patients may lead to a decline in functional capacity, exacerbating frailty and sarcopenia [12]. Therefore, careful consideration of neurological status, electrolyte imbalances, acid-base disturbances, and frailty status is essential when initiating dialysis in these patients. Most older patients on haemodialysis lose functional independence within a year [13], highlighting the need for social support and assistance [14].

Individualized and cautious dialysis approaches are necessary for older patients to prevent an increased risk of dependency and a diminished post-dialysis life. Frail and dependent patients require a treatment that does not further limit their activity levels. Fatigue after dialysis and rapid fluid shifts have been shown to have an impact on overall health and functionality after treatments [15]. Coexisting morbidities such as heart failure, systemic vascular injury, and autonomic dysfunction contribute to intradialytic hypotension, early termination of hemodialysis sessions, and vascular access complications. Survival and quality of life may be limited by multimorbidity, making the management of uremia and dialysis dose less relevant [16].

In this study, we aim to analyze and describe the clinical situation of older hemodialysis patients, with a particular focus on their levels of dependency and frailty. We try to compare subgroups within this patient population based on varying degrees of these characteristics and observe if they influence the prescribed dialysis protocols. The goal is to determine the individualized treatment approaches that may be required and underscore the importance of tailoring hemodialysis treatments to prevent further deterioration in this vulnerable group.

## Methods

### Characteristics of the participants

This study is an observational and descriptive investigation conducted over a year, from January to December 2022. It focused on patients enrolled in the chronic hemodialysis program across four outpatient centers and one hospital unit of the Fundación Renal Íñigo Álvarez de Toledo. Throughout this period, data were collected and analyzed to gain deeper insights into the clinical condition of these patients.

The inclusion criteria were as follows: among all the patients undergoing dialysis in the units, those aged over 75 years, who had been in the programme for more than 3 months and who had accepted and signed the informed consent form were included in the study. 107 subjects were enrolled during this period. There were no exclusion criteria.

### Clinical assessment variables

Demographic and clinical data were obtained from the electronic medical records of the reference centre. The variables considered for the study were: age, sex, height, weight, and body mass index (BMI), aetiology of renal disease, time on haemodialysis, residual diuresis > 500 ml/min and type of vascular access. In relation to the haemodialysis regimen, the variables of duration in hours of the HD session at the start of the programme and at the time of the study, hours per week and days per week were collected. We selected residual diuresis as a criterion because, in dialysis patients, having residual diuresis indicates increased toxin elimination, fewer vascular problems, and a lower incidence of hypotensions.

The analytical data determined were albumin, total iron binding capacity (TIBC) and creatinine. In addition, a measure of dialysis efficacy was established using balanced Daurgides Kt/Ve [17].

Other variables collected were institutionalisation of the patient, type of transfer to the haemodialysis session (own means, ambulance sitting or lying down) and whether or not the patient reported post-treatment fatigue that prevented them from carrying out their usual activities. Post-dialysis fatigue is a concept that is difficult to measure, but we define it as the inability to leave the bed or chair due to lack of strength for more than 24 h after the session, meaning the capacity to overcome the tiredness caused by the dialysis session.

### Measuring tools

#### Comorbidity, as determined by the CHARLSON Index

This is a weighted index that takes into account the number and severity of comorbid illness and assesses the risk of death due to illness. In addition to age, it adds 1 point for each decade of life after the age of 40 and consists of 19 items (with scores from 1 to 6), which, if present, have been shown to influence the subject’s life expectancy in a specific way. Initially adapted to assess survival at one year, it was finally adapted in its final form for survival at 10 years [18]. Several cut-off points have been established, above 3 points it is considered high comorbidity. In dialysis patients, high comorbidity has been considered above 6 points [19]. Some authors have established a significant increase in one-year mortality in those patients with Charlson above 8 points [20], which is why this cut-off point was established as a low differential for high comorbidity.

#### Malnutrition-inflammation scale (MIS)

MIS is a fully quantitative score adopted from a subjective global assessment and is developed for early identification of malnutrition-inflammation states. It is a validated questionnaire for the dialysis population and is composed of 10 components, each scored from 0 to 3: weight change, appetite, gastrointestinal symptoms, functional capacity related to nutritional factors, comorbidities including years on dialysis, subcutaneous fat loss, muscle mass, BMI, serum albumin, total iron binding capacity. The score ranges from 0 to 30 points. Above 10 points we can consider the patient extremely malnourished, 7 to 10 points very severe malnutrition, 5 to 7 points moderate-severe malnutrition, 2 to 5 points mild-moderate malnutrition and less than 2 points would be normonutrition [21].

#### Dependency assessment scale, BARTHEL

To assess the degree of functional dependence, the Barthel index is considered to be the most appropriate scale for assessing basic activities of daily living (Basic ADL), providing a quantitative estimate of the degree of dependence of the person being assessed. Loss of functional capacity is associated with an increased likelihood of institutionalisation, health service use and mortality [22]. BADLs refer to the most basic levels of function, activities such as eating, transferring, dressing and toileting. In the Barthel index, the values assigned to each activity depend on the time taken to perform it and the need for assistance to carry it out. The overall range varies between 0 and 100 points. Below 20 points we have total dependence, from 21 to 60 points severe dependence, from 61 to 90 points moderate dependence, from 91 to 99 points mild dependence and 100 points would determine independence. This test not only provides an overall assessment of functionality but also shows the specific deficiencies in each of the activities, facilitating the assessment of the patient’s evolution [23].

#### Frailty rating scale, FRIED

The FRIED scale, which considers frailty as a phenotype of poor physical function, relies primarily on two objective measures: grip strength and gait speed (physical frailty). The most commonly used scale in this model is the Fried scale, described and validated in the Cardiovascular HealthStudy [24] which defines frailty by the presence of 3 or more of the following characteristics (the presence of 1 or 2 factors is considered a pre-frailty state):

1. weight loss:> 4.5 kg or > 5% in the last year and unintentional.
2. Self-perceived exhaustion: this is declared and identified according to 2 questions from the Center for Epidemiological Studies-Depression (CES-D) questionnaire. We will ask two questions about your last week: -Did you feel most of the time that everything you did was an effort? - Did you feel that you couldn’t go on? The answers can be: (a) rarely or never; (b) 1 or 2 days; (c) 3 or 4 days; (d) most of the time. A response ≥ 2 is considered a positive criterion for frailty.
3. Weakness: maximum digital grip strength with dynamometer adjusted for sex and body mass index (BMI). The patient must be seated, with the dominant hand (in our case, as the strength may be affected by the presence of the AV in the arm, we will take as dominant the highest measurement of the two arms) and the elbow at 90°. The highest value of 3 measurements 1 min apart is considered, (males, BMI ≤ 24: strength ≤ 29; BMI ≤ 28: strength ≤ 30; BMI > 28: strength ≤ 32; females, BMI ≤ 23: strength ≤ 17; BMI 23,1–26: strength ≤ 17,3; BMI 26,1–29: strength ≤ 18; BMI > 29: strength ≤ 21).
4. Gait speed (time to cover 4.6 m at usual pace, adjusted for sex and height). We will mark 1 m in front and 1 m behind the 4.6 m marks, so that the time is not affected by acceleration and deceleration (males height ≤ 173 cm, ≥ 7 s; height > 173 cm, ≥ 6 s; females height ≤ 159 cm, ≥ 7 s; height > 159 cm, ≥ 6 s).
5. Low level of physical activity (weekly energy expenditure in physical activity): males, < 383 kcal/week; females, < 270 kcal/week (corresponds to a number of hours per week of walking or the equivalent of swimming, cycling, tennis, etc.; walking: males, < 2.30 h/week; females, <2 h/week).

#### Quality of life scale, SF12

It is a questionnaire of health-related quality of life. Composed of twelve items, its purpose is to provide an easy-to-use instrument to assess the degree of well-being and functional capacity of people over 14 years of age [25], defining a positive and negative state of physical and mental health, by means of eight dimensions (physical function, physical role, bodily pain, mental health, general health, vitality, social function and emotional role). The response options form Likert-type scales (where the number of options varies from three to six points, depending on the item), which assess intensity and/or frequency of people’s health status. The score ranges from 0 to 100, the higher the score, the better the health-related quality of life, the cut-off is generally established at 50 point to discriminate between high and low quality score. Research using the twelve items of the SF has verified that this instrument is a valid and reliable measure, with significant correlations between versions of the scale [26, 27].

### Ethical considerations

The study was approved by the ethics committee of the Hospital Universitario Fundación Jiménez Díaz on 18/01/2022 (act nº 01/22) and followed the regulations of the European Union law on data protection and privacy for all persons within the European Union (GDPR / 2018), the Declaration of Helsinki on ethical principles for medical research involving human subjects.

### Statistical analysis

The statistical approach was carried out using IBM SPSS Statistics V20. Quantitative variables were presented as mean and standard deviation. Qualitative variables were presented as absolute numbers and percentages.

The and ANOVA were used for comparative analysis between quantitative variables. The association between qualitative variables was assessed using the chi-square test. The level of statistical significance was determined for a p less than or equal to 0.05.

## Results

### Descriptives

In the study, a total of 107 patients undergoing haemodialysis were included. 57% of whom were male. The mean age was 81.3 ± 4.53 years and the length of stay on HD was 51.71 ± 51.04 months. The aetiologies of the renal disease were diabetes mellitus 24 (22.4%), unaffiliated renal disease 25 (23.4%), vascular 25 (23.4%), tubulointerstitial nephritis 7 (6.5%), glomerular 12 (11.2%), polycystic kidney disease 8 (7.5%) and others 6 (5.6%).

Table 1 shows the characteristics of the study population in terms of demographic data, renal disease and HD regimen, anthropometric and analytical data and measurement scales used. Men are dialysed for longer periods of time related to their larger body surface area and have higher blood creatinine values. Regarding the scores on the scales measured, women had less comorbidity. For the rest, there were no differences in the total score, but within the data measured in the FRIED frailty scale, in addition to greater strength and walking speed in men, justifiable by their difference in body composition, women presented less physical activity and in the test measuring quality of life, women presented worse scores in the mental health sphere.

**Table 1:** Demographic data, renal disease and haemodialysis regimen, anthropometric, analytical and comorbidity assessment scales, malnutrition-inflammation, dependence, frailty and quality of life. Mean ± SD or n (%)

	Total *n* = 107 (100%)	Male *n* = 61 (57%)	Female *n* = 46 (43%)	*P*
**Demographic data**				
Age (Years)	81.3 ± 4.535	80.9 ± 4.2	81.8 ± 4.87	0.318
Dialysis vintage (months)	51.71 ± 51.04	43.7 ± 39.6	62.3 ± 61.1	***0.062***
**Aethiology and residual diuresis**				
Diabetes mellitus	24 (22%)	18/24(75%)	6/24 (25%)	***0.086***
Unknown	26 (24%)	15/26(58%)	11/26(42%)	
Vascular	25 (24%)	14/25(56%)	11/25(44%)	
Tubulo-Interstitial Nephritis	6 (7%)	1/6(17%)	5/6(83%)	
Glomerular	12 (11%)	8/12(67%)	4/12(33%)	
Polycystic Kidney Disease	8 (7%)	2/8(25%)	6/8(75%)	
Others	6 (6%)	3/6(50%)	3/6(50%)	
Diuresis > 500 ml/day (Yes)	68(63.6%)	41(67.2%)	27(58.7%)	0.365
**Hemodialysis regiment**				
Time in HD per session (hours)	3.6 ± 0.46	3.8 ± 0.45	3.5 ± 0.47	***0.058***
Time in HD higher or equal to 4 h (Yes)	50/107 (47%)	34/61 (56%)	16/46 (35%)	**0.031**
Time in HD per week > than 12 h (Yes)	49/107 (46%)	33/61 (54%)	16/46 (35%)	**0.047**
**Analytical data**				
Albumin > 3.5 mg/dl	82/107(76.6%)	48/61(79%)	34/46(74%)	0.563
Creatinine (mg/dl)	6.35 ± 1.63	6.67 ± 1.68	5.92 ± 1.47	**0.018**
KTV > 1,3	83/107(85.6%)	46/61(82%)	37/49(90%)	0.262
**Value scales**				
Charlson comorbity (pts.)	9.24 ± 2.02	9.64 ± 2.21	8.72 ± 1.63	**0.019**
MIS nutrition (pts.)	6.85 ± 3.89	6.34 ± 3.61	7.52 ± 4.18	0.121
Barthel dependency (pts.)	74.53 ± 25.38	76.72 ± 25.51	71.63 ± 25.19	0.307
Fried Frailty Total (pts.)	2.33 ± 1.33	2.13 ± 1.23	2.59 ± 1.42	***0.079***
*Fried_weight loss > 4.5Kg (SI)*	6/107(6%)	3/6(50%)	3/6(50%)	0.721
*Fried_low energy (Yes)*	28/107(26%)	12/28(43%)	16/28(57%)	***0.078***
*Fried_Grip strenght(kg)*	20.48 ± 6.65	23.55 ± 6.08	16.32 ± 4.97	**< 0.001**
*Fried_Gait speed 4.6 m(s)*	7.11 ± 3.25	6.35 ± 2.24	8.21 ± 4.10	**0.009**
*Fried_Low physical activity (Yes)*	62/107(60%)	30/62(48%)	32/62(52%)	**0.034**
SF12 Quality of life total (pts.)	31.44 ± 6.17	32.25 ± 5.77	30.37 ± 6.57	0.120
*SF12_mental health(pts.)*	2.99 ± 1.28	2.93 ± 0.96	3.07 ± 1.23	0.555
*SF12_physical function(pts.)*	3.41 ± 1.29	3.54 ± 1.2	3.24 ± 1.32	0.35
*SF12_physical role (pts.)*	2.72 ± 0.82	2.77 ± 0.82	2.65 ± 0.82	0.464
*SF12_body pain(pts.)*	3.34 ± 1.38	3.54 ± 1.36	3.07 ± 1.37	0.077
*SF12 Physical Component Summary (pts.)*	12.45 ± 2.36	12.77 ± 2.57	12.02 ± 3.19	0.182
*SF12_vitality(pts.)*	3.28 ± 1.43	3.25 ± 1.34	3.33 ± 1.55	0.775
*SF12_social function(pts.)*	3.71 ± 1.20	3.74 ± 1.22	3.67 ± 1.19	0.778
*SF12_emotional role(pts.)*	3.50 ± 0.84	3.57 ± 0.69	3.41 ± 1	0.329
*SF12_mental health(pts.)*	8.43 ± 2.04	8.90 ± 2	7.80 ± 1.95	**0.005**
*SF12 Mental Component Summary (pts.)*	18.92 ± 3.96	19.44 ± 3.86	18.±4.03	0.114

TIBC: total iron binding capacity. Classification scales. CHARLSON: Low comorbidity (< 9pts.), High comorbidity (> 9pts.) MISS: Extremely malnourished (> 10pts.); Very severe malnutrition (> 7-10pts.); Moderate-severe malnutrition (> 5-7pts.); Mild-moderate malnutrition (> 2-5pts.); Normonutrition (< 2pts.). BARTHEL: Independent (100pts.); Mild dependence (91-99pts.); Moderate dependence (61-90pts.); Severe dependence (21-60pts.); Total dependence (< 20pts.) FRIED: Non-fragile (0pts.); Pre-fragile (1-2pts.); Frail (> 3pts.). SF-12: High quality of life (> 50pts.), Low quality of life (< 50pts.) Statistical significance: *p* < 0.05, Men Vs Women

Table 2 analyses the situation of the sample taking into account the cut-off points established by the different scales that categorise our population. They were grouped into normal and altered categories. To establish the cut-off points for the different categories we grouped according to the cut-offs of the scale itself in the MIS and BARTHEL and used the median for CHARLSON, median 9 pts (6-16pts), and SF12, median 32 pts (15-46pts). We found that 63(59%) of the patients had high comorbidity, 59(55%) were moderately to extremely malnourished, 22(21%) were dependent, 49(49%) were frail patients and 61(57%) had low quality of life. Comparing again the different groups between men and women, we can see that there are no differences, although there are similar differences in the areas of frailty and quality of life, with women having the highest number of frail patients and the poorest quality of life.

**Table 2:** Percentages in degrees of severity of the comorbidity, malnutrition, dependency and fragility scales

Clasification			Total *n* = 107 (100%)		Male *n* = 61 (57%)	Female *n* = 46 (43%)	*P*
CHARLSON comorbity	Low (Normal)		44/107 (41%)		21/44 (48%)	23/44 (52%)	0.105
	High (Altered)		63/107 (59%)		40/63 (64%)	23/63 (36%)	
MIS desnutrition	Normal	Normonutrition	11/107 (10%)	48/107(45%)	30/48 (63%)	18/48(37%)	0.301
		Mild-moderate malnutrition	37/107 (35%)				
	Altered	Moderate-severe malnutrition	18/107 (17%)	59/107 (55%)	31/59 (53%)	28/59 (47%)	
		Very severe malnutrition	19/107 (18%)				
		Extremely malnourished	22/107 (21%)				
Barthel dependency	Normal	Independent	27/107 (25%)	85/107(79%)	50/85(59%)	35/85 (41%)	0.478
		Mild dependence	58/107 (54%)				
	Altered	Moderate dependence	9/107 (8%)	22/107(21%)	11/22(50%)	11/22(50%)	
		Severe dependence	7/107 (6%)				
		Total dependence	6/107 (5%)				
Frail frailty	Normal	Non-fragile	9/107 (8%)	58/107(54%)	38/58(66%)	20/58(34%)	***0.053***
		Pre-fragile	49/107 (46%)				
	Altered	Frail	49/107 (46%)		23/49(47%)	26/49(53%)	
SF-12 Quality of life	High (Normal)		46/107 (43%)		31/46 (67%)	15/46 (43%)	***0.060***
	Low (Altered)		61/107 (57%)		30/61 (49%)	31/61 (51%)	

Classification scales. CHARLSON: Low comorbidity (< 9pts.), High comorbidity (> 9pts.) MISS: Extremely malnourished (> 10pts.); Very severe malnutrition (> 7-10pts.); Moderate-severe malnutrition (> 5-7pts.); Mild-moderate malnutrition (> 2-5pts.); Normonutrition (< 2pts.). BARTHEL: Independent (100pts.); Mild dependence (91-99pts.); Moderate dependence (61-90pts.); Severe dependence (21-60pts.); Total dependence (< 20pts.) FRIED: Non-fragile (0pts.); Pre-fragile (1-2pts.); Frail (> 3pts.). SF-12: High quality of life (> 32pts), Low quality of life (< 32pts.) Statistical significance: *p* < 0.05, Male Vs Female

As for the rest of the variables recorded, 40 (67%) were dialysed via a catheter. 68 (64%) of the patients urinate and 38 (36%) urinate more than 500 ml per day. Of the total number of patients, 87 (82%) are able to walk and 87 (82%) are transferred to the centre by ambulance and the rest by their own means. Only 4 (4%) are lying on a stretcher due to their state of dependency. 9 (8%) of the patients live at home and up to 60 (56%) of the patients report extreme fatigue after dialysis treatment.

### Dependency and frailty analysis

Table 3 presents the association between dependency, considering the normal/altered cut-offs described in Table 2 and the rest of the variables.

As can be seen in the table, the most dependent patients do not maintain residual diuresis, have higher comorbidity, are the most malnourished and frail. In terms of lifestyle variables, they have difficulty walking, live institutionalised, are transported to the dialysis centre by ambulance while lying down and report feeling very tired after treatment.

**Table 3:** Association between dependence and the rest of the qualitative variables. Data expressed as n (%) or mean ± SD

	Independients (normal), *n* = 85 (79%)	Dependents (altered), *n* = 22 (21%)	*P*
**Demographics, renal disease and HD regimen**			
**Sex**			
Male/Female	50/35(59%)	11/11 (50%)	0.478
**Aetiology**			
Diabetes mellitus, *n* = 24	19/85(22%)	5/22(23%)	0.315
Unknown, *n* = 25	18/85 (21%)	7/22(32%)	
Vascular, *n* = 25	18/85(21%)	7/22(32%)	
Tubular intersticial nephritis, *n* = 7	7/85 (8%)	0/22(0%)	
Glomerular, *n* = 12	12/85(14%)	0/22(0%)	
Polycystic kidney, *n* = 8	7/85(8%)	1/22(5%)	
Others, *n* = 6	4/85 (5%)	4/22(9%)	
**Diuresis Residual diuresis**			
Yes, *n* = 68	60/85 (71%)	8/22 (36%)	**0.003**
**Dialysis hours per week**			
Less than 12 h, *n* = 49	40/85 (47%)	9/22 (41%)	0.606
More than12 hours, *n* = 58	45/85 (53%)	13/22 (55%)	
**Vascular access**			
Arteriovenous fistula, *n* = 57	45/85 (53%)	12/22 (55%)	0.893
Permanent cathether, *n* = 50	40/85 (47%)	10/22 (45%)	
**Analitical data**			
Albumin > 3.5 mg/dl, *n* = 82	68/85 (80%)	14/22 (64%)	0.106
KTV > 1,3, *n* = 83	65/75 (87%)	18/22 (82%)	0.730
**Rating scales**			
**Charlson comorbidity**			
Low comorbidity, *n* = 44	41/85 (48%)	3/22 (14%)	**0.003**
High comorbidity, *n* = 63	44/85 (52%)	19/22 (86%)	
**MIS nutrition**			
Normonourish, *n* = 48	47/85 (55%)	1/22 (4%)	**< 0.001**
Malnourish, *n* = 59	38/85 (45%)	21/22 (96%)	
**Fried Frailty(2 cathegories)**			
No Frail, *n* = 58	55/85 (65%)	3/22 (14%)	**< 0.001**
Frail, *n* = 49	30/85 (35%)	19/22 (86%)	
**SF-12 Quality of life**			
High QoL, *n* = 46	45/85 (53%)	6/22 (27%)	0.095
Low QoL, *n* = 61	40/85 (47%)	16/22 (73%)	
*SF12 Physical Component Summary (pts.)*	12.99 ± 2.57	10.36 ± 3.05	**< 0.001**
*SF12 Mental Component Summary (pts.)*	19.38 ± 3.56	17.14 ± 4.82	**0.017**
**Lifestyle**			
**Ability to walk**			
Yes, *n* = 87	82/85 (97%)	5/22 (23%)	**< 0.001**
**Lives in a retirement home**			
Yes, *n* = 9	4/85 (5%)	5/22 (23%)	**0.007**
**Transport to the HD centre and home**			
Ambulance lying down, *n* = 3	0/85(0%)	3/22 (14%)	**< 0.001**
Ambulance seated, *n* = 82	65/85 (77%)	17/22 (77%)	
Own means, *n* = 22	20/85 (23%)	2/22 (9%)	
**Extreme post-treatment fatigue**			
Yes, *n* = 60	43/85 (51%)	17/22 (77%)	**0.025**

Classification scales. CHARLSON: Low comorbidity (< 9pts.), High comorbidity (> 9pts.) MISS: Extremely malnourished (> 10pts.); Very severe malnutrition (> 7-10pts.); Moderate-severe malnutrition (> 5-7pts.); Mild-moderate malnutrition (> 2-5pts.); Normonutrition (< 2pts.). BARTHEL: Independent (100pts.); Mild dependence (91-99pts.); Moderate dependence (61-90pts.); Severe dependence (21-60pts.); Total dependence (< 20pts.) FRIED: Non-fragile (0pts.); Pre-fragile (1-2pts.); Frail (> 3pts.). SF-12: High quality of life (> 32pts), Low quality of life (< 32pts) Statistical significance: *p* < 0.05

In this study, we aim not only to analyze dependency but also to examine the frailty of these patients. Similarly, the association between frailty following the FRAIL scale and the rest of the variables is shown in Supplementary Table 1. Dependency is the clinical manifestation of frailty. Dependency reveals differences that are not observed with frailty, such as in residual diuresis, Charlson comorbidity, and the need for ambulance transfer, thus dependency more accurately reflects the clinical reality.

### Analysis of the presence of post-dialysis fatigue

Table 4 presents the association between fatigue after treatment and the rest of the variables.

**Table 4:** Association between the presence or absence of post-hemodialysis fatigue and the rest of the qualitative variables. Data expressed n (%) or mean ± SD

	No Post Dialysis Fatigue, *n* = 47 (44%)	Post Dialysis Fatigue, *n* = 60 (56%)	*P*
**Demographics, renal disease and HD regimen**			
**Sex**			
Male, *n* = 61	29/47 (62%)	32/60 (53%)	0.385
**Aetiology**			
Diabetes mellitus, *n* = 24	10/47(21%)	14/60(23%)	0.891
**Residual diuresis**			
Yes, *n* = 68	29/47 (62%)	39/60 (65%)	0.725
**Number of dialysis sessions per week**			
2, *n* = 3	2/47 (4%)	1/60 (2%)	0.493
3, *n* = 103	45/47 (96%)	58/60 (97%)	
5, *n* = 1	0/47 (0%)	1/60 (2%)	
**Dialysis hours per session**			
Less tan 4 h, *n* = 50	21/47 (45%)	29/60 (48%)	0.707
More than 4 h, *n* = 57	26/47 (55%)	31/60 (52%)	
**Horas de diálisis a la semana**			
Less than 12 h, *n* = 49	21/47 (45%)	28/60 (47%)	0.838
More than 12 h, *n* = 58	26/47 (55%)	32/60 (53%)	
**Vascular access**			
Arteriovenous fistula, *n* = 57	30/47 (64%)	27/60 (45%)	***0.053***
Permanent cathether, *n* = 50	17/47 (36%)	43/60 (55%)	
**Analytical data**			
**Albumin**			
Albumin > 3.5 mg/dl	39/47 (83%)	43/60 (72%)	0.170
**KT/V**			
KTV > 1,3, *n* = 83	33/38 (87%)	50/59 (85%)	0.774
**Rating scales**			
**Charlson comorbidity**			
Low comorbidity, *n* = 44	25/47 (53%)	19/60(32%)	**0.025**
Highcomorbidit, *n* = 63	22/ 47(47%)	41/60 (68%)	
**MIS nutrition**			
Normonutrition, *n* = 48	26/47 (55%)	22/60 (37%)	***0.054***
Mild malnutrition, *n* = 59	21/47 (45%)	38/60 (63%)	
**BARTHEL dependency**			
Independient, *n* = 85	42/47(89%)	43/60 (72%)	**0.025**
Dependient, *n* = 22	5/47 (11%)	17/60(28%)	
**Fried Frailty**			
No Frail, *n* = 58	36/47(77%)	22/60 (37%)	**< 0.001**
Frail, *n* = 49	11/47 (23%)	38/60 (63%)	
**SF-12 Quality of Life**			
High QoL, *n* = 46	23/47 (49%)	23/60 (38%)	0.272
Low QoL, *n* = 61	24/47 (51%)	37/60 (62%)	
SF12 Quality of life total (pts.)	13.45 ± 2.45	11.67 ± 2.94	**0.001**
*SF12 Mental Component Summary (pts.)*	19.70 ± 3.3	18.30 ± 4.40	***0.069***
**Lifestyle**			
**Ability to walk**			
Yes, *n* = 87	43/47 (92%)	44/60 (73%)	**0.017**
**Lives in a retirement home**			
Yes, *n* = 9	3/47 (6%)	6/60 (10%)	0.503
**Transport to the HD centre and home**			
Ambulance lying down *n* = 3	0/47 (0%)	3/60 (5%)	0.182
Ambulance seated, *n* = 82	35/47 (75%)	47/60 (78%)	
Own means, *n* = 22	12/47 (25%)	10/60 (17%)	

Classification scales. CHARLSON: Low comorbidity (< 9pts.), High comorbidity (> 9pts.) MISS: Extremely malnourished (> 10pts.); Very severe malnutrition (> 7-10pts.); Moderate-severe malnutrition (> 5-7pts.); Mild-moderate malnutrition (> 2-5pts.); Normonutrition (< 2pts.). BARTHEL: Independent (100pts.); Mild dependence (91-99pts.); Moderate dependence (61-90pts.); Severe dependence (21-60pts.); Total dependence (< 20pts.) FRIED: Non-fragile (0pts.); Pre-fragile (1-2pts.); Frail (> 3pts.). SF-12: High quality of life (> 32pts), Low quality of life (< 32pts) Statistical significance: *p* < 0.05

## Discussion

In summary, our study focused on older patients’ haemodialysis patients and revealed several significant findings. Among the older patient’s population in our study, a substantial proportion experienced various challenges, including malnutrition (55%), dependency (21%), frailty (46%), and a low quality of life (57%). Dependent patients exhibited distinct characteristics compared to independent patients, such as reduced urine output, high comorbidity, severe malnutrition, increased frailty, residence in nursing homes, reliance on ambulance transportation for dialysis, and limited mobility (77% unable to walk). Furthermore, 56% of the patients experienced post-dialysis fatigue, which significantly hindered their ability to lead a normal life. These fatigued patients also displayed higher comorbidity rates, increased dependency, greater frailty, and poorer quality of life. Notably, the dialysis patterns remained consistent across all patient groups, regardless of their clinical condition.

According to the United States Renal Data System, older patients patients aged 75–79 years with end-stage renal disease (ESRD) undergoing haemodialysis (HD) have an expected remaining lifetime of 2.8 years, while those aged 80–84 years and over 85 years have remaining lifetimes of 2.3 years and 1.9 years, respectively [28]. However, our study population had a mean age of 81.3 years and an average dialysis duration of 51.71 months, which exceeded the expected values. However, it’s noteworthy that our study cohort had an average age of 81.3 years and an average duration of dialysis treatment spanning 51.71 months, which significantly surpasses these expected values. While it’s important to recognize that these two populations are not directly comparable due to their inherent differences, our study offers valuable insights into outcomes among similar groups.

Frailty was prevalent among haemodialysis patients, affecting 46% of the older patients. Frailty rates in kidney failure patients vary widely depending on the population and assessment methods, ranging from 30 to 70% [29]. Frailty in dialysis patients is associated with increased risks of falls, hospitalizations, cognitive decline, vascular access failure, and mortality [30].

Functional dependence in the older patients can have negative effects on quality of life, increase caregiver burden, and result in higher healthcare utilization [31]. In our study, 21% of the older patients were dependent, and 56% experienced debilitating fatigue after dialysis, impeding their ability to live a normal life until the next session. These tired patients had higher comorbidity rates, increased dependency, greater frailty, and poorer quality of life, along with reduced mobility. Notably, dialysis schedules did not differ significantly among dependent or fatigued patients compared to non-dependent or non-fatigued individuals [5].

Additionally, our study revealed notable sex differences in frailty, with women exhibiting less physical activity and worse scores in the mental health domain of the quality of life assessment. This suggests that gender-specific factors may play a significant role in the manifestation and impact of frailty among older hemodialysis patients. These findings underscore the need for gender-sensitive approaches in assessing and managing frailty to optimize patient outcomes.

Aggressive end-of-life care is commonly provided to haemodialysis patients and is more intense than for individuals with other chronic life-limiting illnesses [32].

Patients on haemodialysis spend more time in hospitals and in the haemodialysis unit, with a decreased likelihood of dying at home compared to those receiving supportive care. The initiation of dialysis is often accompanied by a decline in functional status within the first six months, particularly among older and frail patients. Caregiver burden also increases during this period [33].

The recovery time following haemodialysis sessions varies widely among patients. Some individuals require more than 12 h to recover, and longer recovery times are associated with older age and comorbidity. Incremental haemodialysis has been shown to reduce recovery time and may be especially beneficial for older patients with limited life expectancy [8].

Therefore, it’s crucial to consider a patient’s existing quality of life and health goals during the predialysis phase [34]. Examples include living at home and participating in social activities. Through physical activity, interventions can be started to stop functional decline [35].

Wide variations in recovery times have been revealed by the Dialysis Outcomes and Practice Patterns Study (DOPPS). 10% of all patients required more than 12 h to recover from an HD session, with longer recovery times being related to comorbidity [36] and advancing age. In centers using incremental HD, patients recovered from their HD session faster, with significantly more patients reporting recovery between 1 and 4 h [37], according to a recent study. Many frail senior patients are left with little time to spend with their families at home because to the longer recuperation time caused by the dependency on transportation to an in-center HD session. Dependents have much higher comorbidity, are more malnourished, frailer and have poorer quality of life. Dependents live longer in residential care, go to dialysis by ambulance and have extreme fatigue at the end of dialysis.

Mortality in patients on conservative treatment who advance to CKD stage 5 and have an eGFR 10 ml/min/1,73m2 is very high. Compared to conservative care, dialysis improves the survival of older patients with ESRD. Age, however, reduces this survival advantage, and some ESRD patients show long-term survival without renal replacement therapy. The time to event study also revealed that patients who live for more than three months had a lower mortality risk [38]. Our population has a time on HD of 51.71 months, which is very long.

Patients with chronic renal disease were more worried about the influence on QoL than longevity, according to a comprehensive review and synthesis of qualitative studies on their opinions on treatment decision-making [39].

Predicting which patients’ functional status will improve after starting dialysis (assumed through improvement of uremic symptoms) and which it will worsen (for example, due to the burden of dialysis therapy) is crucial. A large burden of injury can result with standard dialysis administration [40], despite the potential advantages of a sufficient dose of dialysis being strongly advised. Despite the fact that this is true for all dialysis patients, the consequences may be more obvious in the older patients and fragile [41]. In this study the mean HD session time was 3.6 h and started with 3.39 h session time. KTV > 1.3 was in 85.6% of patients. The mean KTV was very high (1.6), with 83% above 1.3. 21% of our total sample were dependent and of these 64% did not urinate. Of those dependent 55% dialyse more than 12 h/week. There is no difference in either vascular access or dialysis time, although most of the dependent patients do not urinate.

All patient relevant outcomes, including survival from diagnosis, frequency of hospitalizations, number of hospital and ICU days, QoL and symptom load, and eventually the fraction of hospital vs. home deaths, should be considered in older HD patients. For comparative efficacy calculations, it would also be crucial to determine the costs of both treatment modalities [42].

When making treatment decisions for older haemodialysis patients, it is crucial to consider their individual goals and current quality of life. Patients are often more concerned about the impact on their quality of life than on longevity. The delivery of standard thrice-weekly haemodialysis has been associated with harm, and incremental haemodialysis could be a less burdensome treatment option, particularly for older patients patients with short life expectancies [43].

Recognizing and assessing frailty is essential in changing the approach to older haemodialysis patients. Guidelines for dialysis in all age groups, but especially in the older patients, have limited evidence-based recommendations. Individualized, patient-centered therapy that involves shared decision-making between physicians and patients can lead to a more adaptable dialysis regime.

Limitations of the Study: Our multicentric approach, which included 107 patients aged over 75, examined over a year, represents a significant strength, allowing for a comprehensive assessment of various factors impacting quality of life. This dataset highlights the importance of nephrologists in identifying and addressing aspects that may deteriorate the quality of life for elderly patients.

However, the generalizability of our findings may be limited by the sample size and the demographic homogeneity of the participants, potentially restricting the applicability of our results to a broader elderly population. Furthermore, the use of self-reported measures for fatigue introduces the possibility of subjective bias, which could affect the accuracy of these findings.

## Conclusions

Despite the high age of the sample studied, the average time on haemodialysis was over 4 years. The pattern of starting dialysis does not differ from that of maintenance. Comorbidity, frailty, nutrition and dependency are very high. They need a lot of support in transport and residence. One third of patients do not urinate and their dialysis regimen does not differ from those who do. Most importantly, the physical quality of life is poor in dependent patients with extreme post-dialysis fatigue. We must individualise therapies in order to try to reduce dependency, maintain residual renal function, and avoid extreme post-dialysis fatigue.

### Electronic supplementary material

Below is the link to the electronic supplementary material.

Supplementary Material 1

## Data Availability

Anonymized clinical data employed in this study is available upon request to smas@quironsalud.es.
