# Supplementary material for: Dependency and frailty in the older haemodialysis patient
Source: BMC Geriatr. 2024 May 10;24:416. doi: 10.1186/s12877-024-04973-8 (PMC11088105; doi:10.1186/s12877-024-04973-8)
Supplement: Supplementary file 1 — Supplementary Material 1 [file 12877_2024_4973_MOESM1_ESM.docx]

**Supplementary Table 1 . Association between frailty and the rest of the qualitative variables.**

**Data expressed as n (%) or mean±SD.**

|  | | **Non-fragile, n=9 (8.4%)** | **Prefragile, n=49 (46%)** | **Fragile, n=49 (46%)** | **P** |
| --- | --- | --- | --- | --- | --- |
|  | ***Demographics, renal disease and HD regimen*** | | | | |
|  | ***Sex*** | | | | |
| **Male/Female** | | 4/5(44%) | 34/15 (69%) | 23/26 (47%) | **0.05** |
|  | ***Aetiology*** | | | | |
| **Diabetes mellitus, n=24** | | 3/9(33%) | 10/46(21%) | 11/46(22%) | 0.315 |
| **Unknown, n=25** | | 18/85 (21%) |  | 7/22(32%) |  |
| **Vascular, n=25** | | 18/85(21%) |  | 7/22(32%) |  |
| **Tubular intersticial nephritis, n=7** | | 7/85 (8%) |  | 0/22(0%) |  |
| **Glomerular,n=12** | | 12/85(14%) |  | 0/22(0%) |  |
| **Polycystic kidney, n=8** | | 7/85(8%) |  | 1/22(5%) |  |
| **Others, n=6** | | 4/85 (5%) |  | 4/22(9%) |  |
|  | ***Diuresis Residual diuresis*** | | | | |
| **Yes** | | 7/9 (78%) | 32/46 (65%) | 29/46 (59%) | 0.59 |
|  | ***Dialysis hours per week*** | | | | |
| **Less than 12 hours,** | | 3/9 (33%) | 22/49 (45%) | 24/49 (49%) | 0.71 |
| **More than12 hours,** | | 6/9 (67%) | 27/49 (55%) | 25/49 (51%) |  |
|  | ***Vascular access*** | | | | |
| **Arteriovenous fistula, n=57** | | 8 (89%) | 26 (53%) | 23 (47%) | 0.071 |
| **Permanent cathether, n=50** | | 1 (11%) | 23 (47%) | 26 (53%) |  |
|  | ***Analitical data*** | | | | |
| **Albumin >3.5 mg/dl,** | | 3/9 (47%) | 22/46 (45%) | 24/46 (49%) | 0.33 |
| **KTV >1,3, n=83** | | 7/7 (100%) | 37/40 (88%) | 39/45 (81%) | 0.55 |
|  | ***Rating scales*** | | | | |
|  | ***Charlson _comorbidity*** | | | | |
| **Low comorbidity, n=44** | | 6/9 (67%) | 23 (47%) | 15 (31%) | 0.079 |
| **High comorbidity, n=63** | | 3/9 (33%) | 26 (53%) | 34 (69%) |  |
|  | ***MIS nutrition*** | | | | |
| **Normonourish, n=48** | | 8/9 (89%) | 29/49 (59%) | 11 (22%) | **<0.001** |
| **Malnourish, n=59** | | 1/9 (11%) | 20/49 (41%) | 38 (78%) |  |
|  | ***Barthel_Dependence*** | | | | |
| **No Dependent, n=85** | | 9/9 (100%) | 46/49 (94%) | 30/49 (61%) | **<0.001** |
| **Dependent, n= 22** | | 0/9 (0%) | 3/49 (6.1%) | 19/49 (39%) |  |
|  | ***SF-12 Quality of life*** | | | | |
| **High QoL, n=0** | | 0/9 (0%) | 0/49 (0%) | 0/49 (0%) |  |
| **Low QoL, n= 107** | | 9/9 (44%) | 49/49 (22%) | 49/49 (27%) |  |
| ***SF12 Physical Component Summary (pts.)*** | | 14 (14, 17) | 13 (12, 15) | 12 (9, 13) | **<0.001** |
| ***SF12 Mental Component Summary (pts.)*** | | 21 (18,24) | 19 (18,22) | 17 (15,20) | **0.001** |
|  | ***Lifestyle*** | | | | |
|  | ***Ability to walk*** | | | | |
| **No, n=87** | | 0/9 (0%) | 3/49 (6.1%) | 17/49 (35%) | **<0.001** |
|  | ***Lives in a retirement home*** | | | | |
| **Yes, n=9** | | 2/9 (5%) | 0/49 (0%) | 7/49 (14%) | **0.006** |
|  | ***Transport to the HD centre and home*** | | | | |
| **Ambulance lying down,n=4** | | 0/9(0%) | 1/49(2%) | 3/49 (6.1%) | 0.83 |
| **Ambulance seated, n=83** | | 7/9 (77%) | 38/49 (78%) | 38/49 (78%) |  |
| **Own means, n=20** | | 2/9 (22%) | 10/49 (20%) | 8/49 (16%) |  |
|  | ***Extreme post-treatment fatigue*** | | | | |
| **Yes, n=50** | | 1/9 (11%) | 23/49 (47%) | 26/49 (53%) | **<0.001** |
